# The molecular mechanisms of light adaption in light-harvesting complexes of purple bacteria revealed by a multiscale modeling†
†Electronic supplementary information (ESI) available: RMSD analysis of the MDs; electronic coupling map; averaged absorption spectra of the three complexes; contributions to the BChl transitions from all close residues; functional benchmark on the H-bonded residue contribution; distribution of the *V*1αβ components along the MD; complete excitonic parameters; charge-transfer parameters; excitonic energies; dihedral angle values on optimized structures; site energies from constrained geometry optimizations. See DOI: 10.1039/c9sc02886b


**DOI:** 10.1039/c9sc02886b

**Published:** 2019-09-27

**Authors:** Felipe Cardoso Ramos, Michele Nottoli, Lorenzo Cupellini, Benedetta Mennucci

**Affiliations:** a Dipartimento di Chimica e Chimica Industriale , Università di Pisa , Via G. Moruzzi 13 , 56124 Pisa , Italy . Email: benedetta.mennucci@unipi.it

## Abstract

The light-harvesting in photosynthetic purple bacteria can be tuned in response to the light conditions during cell growth. One of the used strategies is to change the energy of the excitons in the major fight-harvesting complex, commonly known as LH2. In the present study we report the first systematic investigation of the microscopic origin of the exciton tuning using three complexes, namely the common (high-light) and the low-light forms of LH2 from *Rps. acidophila* plus a third complex analogous to the PucD complex from *Rps. palustris*. The study is based on the combination of classical molecular dynamics of each complex in a lipid membrane and excitonic calculations based on a multiscale quantum mechanics/molecular mechanics approach including a polarizable embedding. From the comparative analysis, it comes out that the mechanisms that govern the adaptation of the complex to different light conditions use the different H-bonding environment around the bacteriochlorophyll pigments to dynamically control both internal and inter-pigment degrees of freedom. While the former have a large effect on the site energies, the latter significantly change the electronic couplings, but only the combination of the two effects can fully reproduce the tuning of the final excitons and explain the observed spectroscopic differences.

## Abstract 2

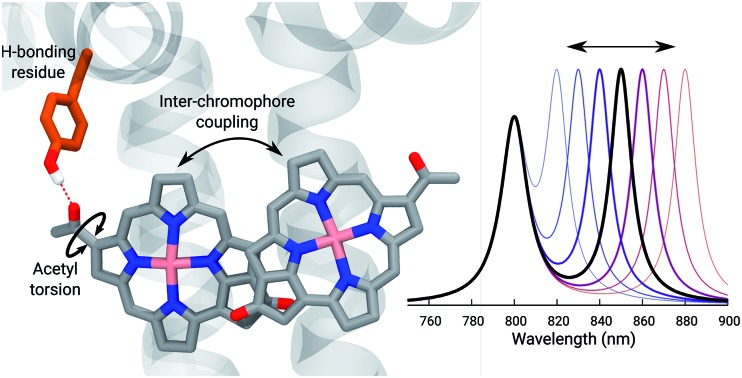
The spectral tuning of LH2 antenna complexes arises from H-bonding, acetyl torsion, and inter-chromophore couplings.

## 1 Introduction

Purple bacteria are photosynthetic organisms with a unique light-harvesting (LH) apparatus, which involves two types of pigment–protein complexes, the so called LH2 and LH1.^1^–^4^ In the photosynthetic membranes, the LH1 complexes surround the reaction center (RC), while the LH2 complexes are arranged more peripherally around the LH1–RC complex; the ratio of the LH2 complexes to LH1–RC complexes is regulated by the incident light intensity.^5^,^6^ Moreover, in some species of purple bacteria, an additional adaptation to changing light conditions is possible thanks to the modular composition of the LH complexes. In LH2 complexes, for example, the units consist of pairs of hydrophobic, low-molecular-weight polypeptides, called α and β: by expressing different genes that encode such polypeptides, different LH2 complexes are obtained showing different spectroscopic properties in the NIR spectral range.^7^–^9^ The α and β chains, in fact, noncovalently bind a number of bacteriochlorophylls a (BChl) which present a bright excitation (the so-called *Q*_y_ excitation) at around 790 nm when isolated, and in the range 800–890 nm when arranged in the multichromophoric aggregates present in the different forms of LH2 and LH1 complexes. These shifts to longer wavelengths are due to excitonic interactions among the *Q*_y_ excitations of the BChls which, in all complexes, are arranged in circular, elliptical, or horseshoe aggregates (from now called rings) presenting different dimensions and symmetries.

**Fig. 1:**
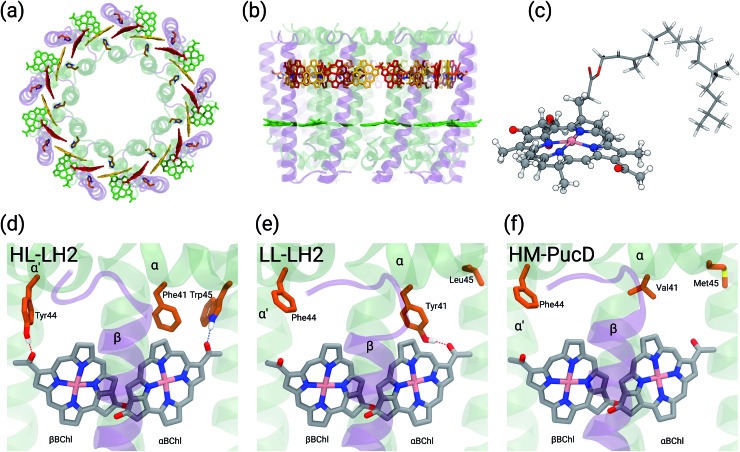
(a) and (b) Structure of LH2 complex from *Rps. acidophila*. The LH2 complexes are circular structures formed by nine dimeric units, the α and β chains, binding one catotenoid molecule (not shown) and three bacteriochlorophyll a (BChl) molecules (*α* and *β*BChl, and B800). (c) Representation of the *β*BChl extracted from the LL–LH2 crystal structure. The atoms represented as spheres were included in the QM part in the multiscale calculations (see Methods), whereas the other atoms were described with the MMPol method. (d, e and f) Schematic representation of the local protein environment for *α* and *β*BChl within the same *αβ* unit inside the three complexes: HL–LH2, LL–LH2 and HM–PucD, respectively.

In particular, one of the species showing different forms of LH2 at different light conditions, the *Rps. acidophila*, is characterized by a nonameric circular symmetry which corresponds to two absorption bands centered at 800 and 850 nm when the cells are grown under high light (HL) conditions. The same organism, when grown under low light (LL) conditions, presents a different form of LH2, whose longer wavelength band is blue-shifted at 820 nm while the other band remains unshifted. Moreover, the shifted band is significantly broadened and less intense. This different spectroscopic form of LH2 is also known in the literature as B800–B820 or LH3. The available crystallographic data clearly show that the HL LH2 (from now on HL–LH2)^10^,^11^ and its LL analog (from now on LL–LH2)^12^ present exactly the same multichromophoric structure with a nonameric symmetric repetition of the basic building block, the αβ unit which accommodates three BChl pigments (and one carotenoid molecule). As a result, two circular rings, made of 9 and 18 BChls respectively, are obtained (Fig. 1a and b). The only significant differences are due to the different local environment around the BChls of the 18-meric ring. In particular, the protein residues adjacent to the BChls in HL–LH2, Tyr44 and Trp45, in LL–LH2 are replaced by phenylalanine and leucine, respectively. While the former ones form hydrogen bonds with the acetyl moiety of the BChl, the latter ones do not. It has also to be noted that in LL–LH2 a new hydrogen bond is however activated from the acetyl group to another tyrosine (Tyr41). This hydrogen bond is not present in the HL–LH2 complex where the residue at the equivalent position is phenylalanine. These changes in the H-bonding patterns between the two complexes are shown in Fig. 1d–f.

Other studies have showed that another purple bacteria species (*Rps. palustris*), when grown in LL conditions, can express different forms of LH2 with multiple gene pairs encoding the LH2 apoproteins.^2^,^13^–^15^ It was suggested that LL LH2 complexes of *Rps. palustris* have a heterogeneous peptide composition,^14^ whereas Papiz and co-workers suggested that these complexes mainly contain the *puc*BA_d_ gene pair sequence.^13^ Recently, a quadruple deletion mutant, containing only the *puc*BA_d_ gene pair, was characterized. The LH2 complex produced by this mutant, called PucD, presents an absorption spectrum with a single band and a strong circular dichroism signal in the 800 nm region, similar to the wild type LL LH2 of *Rps. palustris*.^15^ Unfortunately, such studies were not able to generate a high resolution structural model and different structures have been proposed. In the study by Papiz and co-workers,^13^ the electron density at 7.5 Å resolution of the LL grown LH2 complex of *Rps. palustris* suggests a larger pigment density than what found in the LH2 complexes from *Rps. acidophila*, and an octameric structure with exact 8-fold rotational symmetry. On the contrary, the PucD mutant presents a similar pigment composition to the LH2 complexes of *Rps. acidophila* and a similar nonameric structure, the main differences being once more in the local environment of the BChls.^15^

Many experimental and theoretical studies have tried to identify the origin of the spectroscopic specificities of the different complexes but none of them has yet given a conclusive answer. From the available structural data some hypotheses have been proposed; which can be summarized as follows:

• Hydrogen bonding: hydrogen bonds to the acetyl group conjugated with the BChl macrocycle ring is expected to cause a red-shift in the *Q*_y_ excitation relative to the non-hydrogen bonded case. Hence, the loss of the hydrogen bond in the BChls of LL–LH2 may be the origin of the overall blue-shift.^16^,^17^

• Rotation of the acetyl group: when the acetyl group is in the plane of the BChl macrocycle ring, it adds one more double bond to the conjugated system and the *Q*_y_ excitation is red-shifted. Due to the different H-bonding, the acetyl groups are rotated further out of the plane in the BChls of LL–LH2 and this may be the origin of the overall blue-shift.^12^,^18^,^19^

• Deformation of the structure of the macrocycle: none of the macrocycle rings of the BChl molecules in LH2 are completely planar in the available crystal structures. The BChls of the 9-meric ring are slightly domed, while the ones in the 18-meric ring are differently distorted (see Fig. 1c where the bowed *β*BChl is shown). Deformation of the macrocycle ring has been suggested to give large shifts of the *Q*_y_ absorption band.^20^

• Electrostatic and polarization effects: the residues around the BChls can produce large shifts in absorption, with the magnitude and direction of the shift depending upon the electric fields and the polarization effects due to their specific nature and the disposition in the different complexes.^21^,^22^

• Charge transfer (CT) effects: the coupling between higher energy CT states between BChls in the 18-meric ring and the locally excited *Q*_y_ states influences the exciton structure of the complex,^23^,^24^ lowering the energy of the first bright exciton state, which gives rise to the longest wavelength band in the absorption spectrum. Our recent computational study suggested that the structural differences between the two complexes correspond to a reduction of the CT–*Q*_y_ coupling values in LL–LH2 with respect to HL–LH2.^25^

In the present systematic study we have tested each of the proposed hypotheses by analyzing the LL and HL–LH2 complexes from *Rps. acidophila,* as well as an artificial complex analogous to the PucD complex from *Rps. palustris* which has been predicted by homology modeling (HM–PucD). The latter, despite having the polypeptide sequences of PucD, was built based on the same nonameric structure of LH2 from *Rps. acidophila*. The resulting complex is characterized by a further reduction of H-bonds with respect to LL–LH2, as the Tyr41, which is H-bonded to *α*BChl in LL–LH2, is now replaced by a Valine (see Fig. 1d–f for a comparison of the different hydrogen-bonding pattern in the three LH2 complexes). For the three complexes we have performed classical molecular dynamics (MD) in a lipid membrane to generate equilibrated systems and include the effects of thermal fluctuations. The structures coming from the MD trajectories have then been used to construct the excitonic Hamiltonian to generate the exciton states and the final absorption spectra. All excitonic calculations have used a hybrid quantum mechanics/molecular mechanics approach including a polarizable embedding (QM/MMPol).

The results reproduce the blue-shift of the excitons of the 18-meric ring going from HL to LL–LH2, in agreement with spectroscopic data, while they fail to give the expected further shift from LL–LH2 to PucD. By identifying and quantifying the reasons of the successes and the limits of the adopted computational strategy, an explanation of the mechanisms that govern the adaptation to different light conditions is suggested in terms of a delicate interplay between the H-bonding network around the 18-meric ring and “intra” and “inter” pigment mobility. This explanation opens a new scenario for a structure-based mutagenesis strategy which has the goal to control the relative energy of the excitons.

## 2 Computational details

### 2.1 Structures

The initial structure for the *Rps. acidophila* LH2 complexes were obtained from the Protein Data Bank (PDB): entries ; 1NKZ 11 for the HL–LH2 and ; 1IJD 12 for the LL–LH2.

For the PucD it was necessary to employ a structural prediction process based on a homology modeling (HM) approach.^26^ The sequences for α and β chains of the PucD complex were obtained from GenPept at National Center for Biotechnology Information (NCBI): entries WP_011158559 and WP_011158560, respectively. Due to the larger overall sequence identity with LL–LH2 (70% and 64% for chains α and β, respectively) than with HL–LH2 (57% and 64%) we used the crystal structure of LL–LH2 as template. Firstly, we produced a 2D alignment between each target sequence and its respective template, and then generated 10 homology models for each chain (α and β) of the PucD complex. Next, the models were evaluated based on both their stereochemical quality and structural overlap with the crystal structure; the best model for each chain was selected and used to construct the PucD αβ-apoproteins. To avoid disrupting protein–cofactor interactions, the vicinity of conserved interacting residues was built by inserting mutant fragments taken from the predicted model into the crystal structure of LL–LH2. The N-terminal end of chain α and the C-terminal end of chain β are supposed to be flexible and non-structured; thus, we adjusted some torsions in order to prevent close contacts between monomers in the final nonameric complex. Finally, the α and β chains were assembled with the cofactors (BChls and Cars) from LL–LH2 complex and the C9 symmetry was applied to the system to obtain the entire PucD structure.

### 2.2 Molecular dynamics

To reproduce the membrane environment, we performed molecular dynamics simulations of the three different forms of LH2 in 1,2-dioleoyl-*sn*-glycero-3-phosphocholine (DOPC) bilayers. Using the input-generator tool of CHARMM-GUI server,^27^ we built a DOPC membrane with about 800 lipid molecules in total, solvated with a water layer of 40 Å on both sides and at 0.1 M NaCl. The membrane was then preequilibrated by applying the same procedure used by Dickson *et al.*^28^ The next step was to insert the complexes into the preequilibrated membrane, and to eliminate the close contacts by deleting all lipid molecules up to 1.0 Å from the external part of the complex (the lipid core was preserved). Then, by using the *tleap* module of AmberTools,^29^ we added extra Na^+^ ions so as to achieve system charge neutrality. The final systems contained approximately 290 000 atoms with the simulation box of dimensions approximately 171 Å × 165 Å × 108 Å. The minimization was done by first minimizing the lipid core molecules, next all the lipid tails and finally the whole system.

For the MD simulations we used a protocol very similar to that employed in our previous work.^30^ Briefly, we first performed a heating from 0 to 100 K (5 ps in the NVT ensemble) constraining all the system but not the lipid tails with a harmonic potential (10.0 kcal mol^–1^ Å^–1^) and then from 100 to 300 K (100 ps in the NPT ensemble) constraining just the protein and cofactors. Next, a 10 ns NTP equilibration at 300 K was performed initially applying the same constraints on the protein and cofactors but releasing the harmonic force constant by 1 kcal mol^–1^ Å^–1^ ns^–1^. Finally, a production step of 200 ns at 300 K in the NTP ensemble was performed for each system. In all MD simulations, the time step was set to 2 fs. For system temperature and pressure control we employed a Langevin thermostat and an anisotropic barostat, both implemented in the Amber16. The particle-mesh Ewald algorithm^31^ was used to describe the long-range electrostatic interactions. The MD analysis was performed by using both the *cpptraj*^32^ module of AmberTools and locally developed tools. For trajectories visualization we employed the Visual Molecular Dynamics (VMD) software.^33^

Both minimization and MD simulations were performed using the Amber16 program employing the *ff14SB*^34^ force field for protein and *lipid14* (ref. 28) for lipids. The parameters for the BChls were taken from the literature^35^ and for carotenoids we applied a DFT-based strategy developed in our group, previously described by Prandi *et al.*^36^ Water molecules were described by the TIP3P model.

### 2.3 Excitonic states

We described the excitons of the multichromophoric system as linear combinations of locally excited (LE) states and charge-transfer (CT) states. For each BChl, its lowest and bright excitation (*Q*_y_) has been considered.

The BChls in the 18-meric ring are usually labeled as either α or β, depending on the noncovalent binding to the α or β chain, respectively and they alternate in the ring structure. For the CT states we considered all the possible charge transfers between BChls in adjacent αβ pairs of the ring, so that, for every pair, there are two possible transfers: α → β or β → α. By combining the local excitations and the CT states, the following Hamiltonian can be obtained:
1

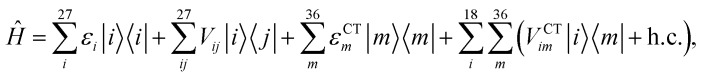

where the indices *i* and *j* run on the locally excited states, *ε*_*i*_ is the excitation energy of the *i*-th BChl and *V*_*ij*_ is the electronic coupling between the *i*-th and *j*-th excitations. The index m runs on the CT states, *ε*CT*m* is the energy of the *m*-th CT state and *V*CT*im* is the coupling between the *i*-th locally excited state and *m*-th CT state. The excitonic analysis was performed by using the EXAT program.^37^

### 2.4 Site energies and couplings

From the MD trajectories, we extracted 50 equally spaced frames from 100 to 200 ns, and for each of them, we computed the site energy (*Q*_y_ state) for the 27 BChls and the corresponding electronic couplings using the QM/MMPol multiscale method.^38^ This method describes the BChl(s) of interest with a QM method and the rest of the environment as a set of classical point charges and atomic polarizabilities. As such, this approach allows the QM part and the classical part to mutually polarize. For the QM part, we used time dependent density functional theory (TD-DFT) at the B3LYP/6-31+G(d) level;^39^,^40^ for the classical part we used the Wang force field.^41^ We excluded the phytyl tail of the BChl from the QM part as it does not influence the *Q*_y_ transition, but we account for its electrostatic and polarization effects describing its atoms as polarizable MM sites (the QM/MM cut is shown in Fig. 1c).

We computed the electronic couplings using a method based on the transition densities of the interacting BChls.^38^,^42^,^43^ That is, for each pair of BChls we computed the Coulomb interaction as
2

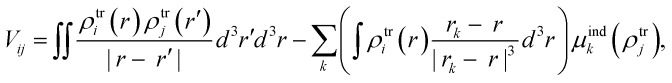

where *ρ*tr*i* and *ρ*tr*j* are the transition density of the *i*-th and *j*-th interacting pigments. The sum in the second term runs on the polarizable sites located at the *r*_*k*_ positions, where *μ*ind*k*(*ρ*tr*j*) is the dipole induced by the transition of the *j*-th pigment. The first and second terms of eqn (2) are respectively the bare Coulomb coupling between the pigments (*V*_Coul_) and the effect of the environment (*V*_MMPol_).

### 2.5 Coupling to charge-transfer states

We computed the effect of CT states with the method proposed in our previous works.^25^,^44^ From 10 of the 50 MD frames, we selected all the possible pairs of adjacent BChls, divided among the intra-chain and inter-chain dimers, for a total of 90 calculations for each dimer. The phytyl tail of each BChl was excluded from the QM part. All couplings within each dimer are computed with the multi-FED-FCD diabatization scheme devised in our previous work,^25^ which combines the Fragment Excitation Difference (FED)^45^ and Fragment Charge Difference (FCD)^46^,^47^ methods. Using appropriate additional operators, the adiabatic Hamiltonian of the dimer is transformed into a diabatic basis, in order to subsequently extract the LE–LE and LE–CT couplings from the diabatic Hamiltonian matrix. The CT energies are corrected *a posteriori* with the corrected Linear Response (cLR) formalism to account for the state-specific response of the environment, which is needed when a large density redistribution upon excitation occurs.

As here we have to compute both LE and CT states, the dimeric calculations were performed with a tuned (*ω* = 0.195) long-range corrected BLYP functional^48^ and the 6-31G(d) basis set. The robustness of this approach with respect to basis sets, functionals, polarization and charge cutoffs has been already validated in our previous work.^44^ The energy of the LC-BLYP/6-31G(d) results was decreased by 1008 cm^–1^ to match the locally excited states found with B3LYP/6-31+G(d). For HM–PucD we used the LL–LH2 CT results as we expect them to be very similar.

### 2.6 Geometry optimizations

In addition to MD simulations we have also performed some tests on the crystal structures. In these cases, due to the limitation in the resolution of the crystal structures, the geometry of the BChls was optimized with a QM/MM method within the ONIOM scheme.^49^,^50^ For the QM BChl, we used the B3LYP/6-31G(d) level and for the MM part, we used the AMBER force field. The BChl phytyl tail was included entirely in the QM part. To be consistent with the previous work,^25^ for HL–LH2 we have used the high-resolution X-ray structure of *Rps. acidophila* determined by Roszak *et al.* (unpublished results) instead of the crystal structure used as a starting point for the MD (PDB entry ; 1NKZ).^11^ The optimization was repeated for both *α* and *β*BChl of HL and LL–LH2 by keeping all the rest frozen, moreover we also froze all the dihedral coordinates of the BChl at their crystal values.

## 3 The picture from the combined MD and QM/MMPol approach

As described in the Introduction, the three LH complexes have been investigated through a combination of classical MD simulations and excitonic QM/MMPol calculations.

In Table 1 we summarize a selection of the calculated excitonic parameters obtained as averages over the configurations coming from the MD simulations of the three complexes. The complete set of parameters is reported in Table S1 of the ESI.†

**Table 1:** Average site energies of the three not equivalent BChls and the two largest couplings within the 18-meric ring. Here *V*1αβ and *V*2αβ indicate the coupling between adjacent BChls belonging to chains of different units (inter-chain), and of the same unit (intra-chain), respectively. All values (in cm^–1^) are reported together with the relative standard deviations computed along 50 frames of the MD trajectories

	HL–LH2	LL–LH2	HM–PucD
*α*BChl	13 527 (276)	13 639 (290)	13 790 (266)
*β*BChl	13 556 (279)	13 693 (297)	13 715 (286)
B800–BChl	13 783 (326)	13 735 (312)	13 767 (328)
*V* 1 αβ	266 (55)	149 (89)	127 (74)
*V* 2 αβ	298 (35)	281 (37)	285 (35)

As expected, the site energies of the BChl in the 9-meric ring (B800) remain almost identical in all complexes. On the contrary, significant changes are found in the *α* and *β*BChls of the 18-meric ring where a blue-shift is obtained moving from HL to LL and HM–PucD. In particular, the excitation of *α*BChl shows a blue-shift of 112 cm^–1^ going from HL to LL and a further shift of 151 cm^–1^ when moving to HM–PucD. In *β*BChl instead, the blue-shift is 127 cm^–1^ from HL to LL, but only 32 cm^–1^ when moving to HM–PucD. As it will be better detailed in the next section, these shifts are clearly correlated with the change in the H-bonding patterns: in fact, moving from HL to LL, *α*BChl loses a H-bond with Trp45, but it gains a H-bond with Tyr41, while *β*BChl loses the H-bond with Tyr44. Further moving from LL–LH2 to HM–PucD, *α*BChl loses the H-bond which this time is not replaced, whereas *β*BChl does not significantly change its local environment.

Also the couplings show significant differences in the three complexes. In particular, the inter-chain *V*1αβ is reduced of *ca.* 44% when moving from HL to LL whereas for the intra-chain analog (*V*2αβ) the reduction is only of 5%; we note that the same trend was previously found by Montemayor *et al.*,^17^ and also suggested from experiments.^22^,^51^ It is also worth noting that virtually no differences are found between LL–LH2 and HM–PucD. The large difference in *V*1αβ arises from the Coulomb component of the coupling, which decreases from HL–LH2 to LL–LH2 and HM–PucD, whereas the effect due to the polarizable environment is similar in the three complexes (Fig. S6 of the ESI†). To further investigate the origin of this difference, we computed two parameters which are expected to affect the coupling, namely the BChl–BChl distance and their mutual orientation (here quantified in terms of the orientation factor *κ* from Förster theory).

**Fig. 2:**
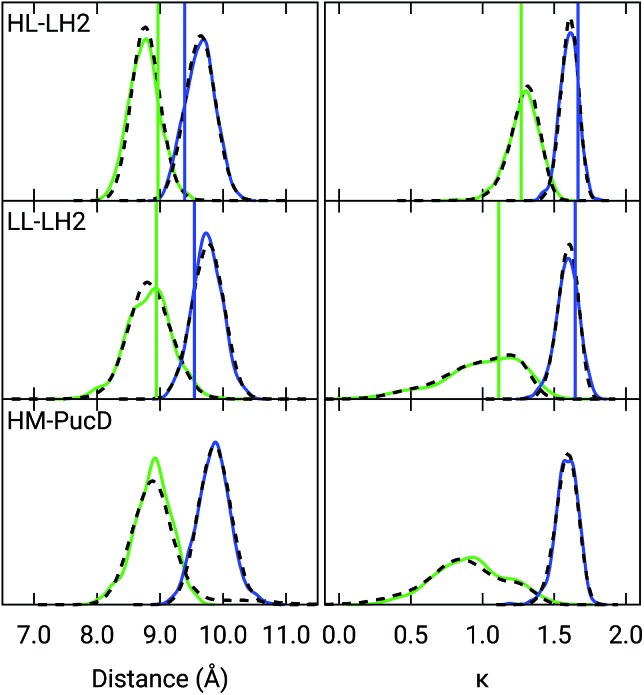
Distribution of the BChl–BChl distance (left) and the orientation factor *κ* (right) for the *V*1αβ (green) and *V*2αβ (blue) couplings for the three complexes. The BChl–BChl distance is here calculated on the basis of the effective center of the macrocycle ring defined as the average of the four nitrogen atom positions. The orientation factor *κ* is defined according to the Förster theory. We plotted the distribution over the whole MD (dashed black line) and over the select 50 frames (colored solid lines). The vertical sticks indicate the values measured on the crystal structures.

As shown in Fig. 2, for the inter-chain pairs (corresponding to the *V*1αβ coupling), the distance remains the same moving from the HL–LH2 to the other two complexes; on the contrary, the mutual orientation changes, becoming less favorable for a large coupling in both LL–LH2 and HM–PucD. For the intra-chain pairs (coupled through *V*2αβ), instead, both distance and mutual orientation remain the same in all complexes. These observations lead to the following conclusion: the adjacent *α* and *β*BChls within the same unit have correlated fluctuations regardless of their H-bond network, while the fluctuations of *α* and *β*BChls across the two units (*i.e.* the inter-chain pairs) are correlated only in the HL–LH2 system, where there is a H-bond connecting the *β*BChl to the neighboring unit (Fig. 1d).

Let us now analyze how these changes in the excitonic parameters are reflected in the exciton states and in the final absorption spectra.

For the sake of clarity, we recall that, in a perfectly symmetric 18-meric cyclic aggregate, the collective exciton states that originate from the coupled *Q*_y_ transitions of individual BChls form a manifold of 18 states where there are two non-degenerate and eight pairwise degenerate excitons. The structure of the aggregates showing transition dipoles of individual BChls almost in the ring plane and the selection rules determine that only the lowest and the highest state pairs (commonly assigned with the quantum numbers *k* = ± 1 and *k* = ± 8, respectively) can be optically excited. The remaining 14 states are dark, *i.e.* not accessible optically from the ground state. Within this picture, an important measurable quantity is the so-called exciton width, namely the difference between the ±8 and ±1 states.^51^–^53^ Static (as well as dynamic) disorder relaxes these symmetry-controlled selection rules by randomly shifting the states and removing their degeneracy. Moreover, disorder affects the exciton dipole strengths allowing their redistribution from optically allowed states of symmetric aggregates (*k* = ± 1 and *k* = ± 8) to adjacent dark states. This is exactly what happens in our simulations based on room temperature MD trajectories; however, it is still possible to define an exciton width by averaging the exciton Hamiltonian over the MD trajectory, and averaging all the site energies and couplings that are equivalent by symmetry. In this way, we obtain the properties of the (average) perfectly homogeneous rings. The resulting exciton widths, together with the shift of *k* ± 1 exciton energy with respect to HL–LH2, are reported in Table 2 and compared with experimental data where available.

**Table 2:** Calculated and experimental exciton widths for the three investigated systems at room temperature and shift of *k* ± 1 exciton energy with respect to HL–LH2. The calculated values reported in square parenthesis are obtained with the inclusion of CT states. The exciton width is the difference between the ±8 and ±1 states of the 18-meric ring. The experimental exciton width for HL–LH2 is from ref. 52. All values are in cm^–1^

		Exciton width	Shift
HL–LH2	Calc	1098 [1179]	—
	Exp	1259	—
LL–LH2	Calc	856 [899]	277 [312]
	Exp	N.A.	501
HM–PucD	Calc	842 [888]	352 [386]
	Exp	N.A.	806

**Fig. 3:**
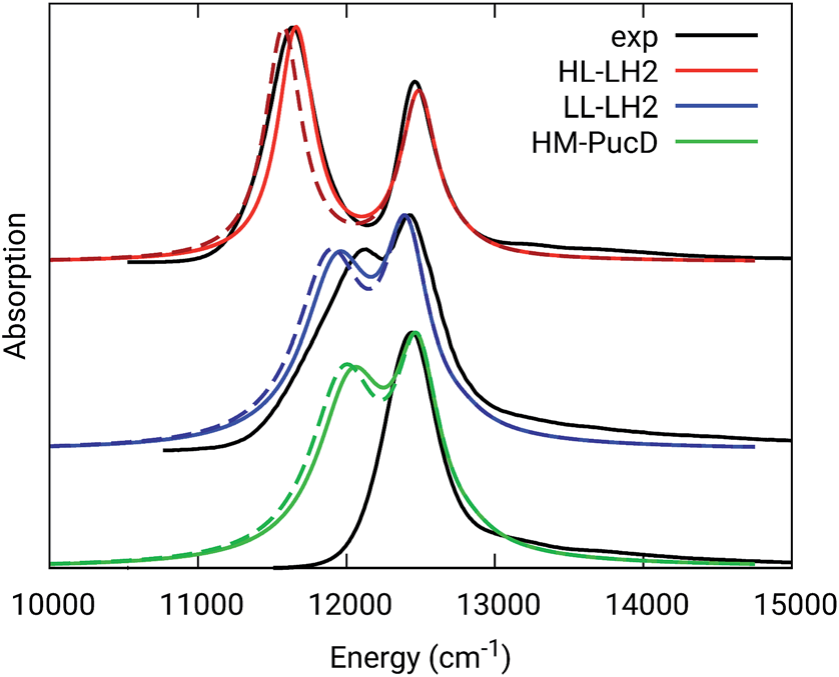
Comparison between simulated and experimental absorption spectra of the three investigated complexes. The spectra computed with the inclusion of CT states are drawn as dashed lines. The experimental spectra for the HL and LL–LH2s have been adapted from ref. 54 while the PucD spectrum has been adapted from ref. 15. In the plots, we shifted all the simulated spectra by –1247 cm^–1^ to match the experimental B800 band: this shift is consistent with the typical error of the QM model (here TDDFT) in describing excitation energies. The lineshape was simulated using a convolution of Lorentzian functions centered on the excitonic states, the widths of the Lorentzian functions were selected to match the experimental data.

The simulated and experimental spectra are reported in Fig. 3 and the energies of the bright excitonic states are reported in Table S3 of the ESI.† The lineshape was simulated as a convolution of Lorentzian functions centered on the exciton energies, whose widths were selected to match the experimental data. More specifically, we used the following half-width at half-maximum (HWHM): HL–LH2 150 cm^–1^, LL–LH2 and HM–PucD 280 cm^–1^ for the low energy band, and 180 cm^–1^ for the high energy band. We note that with this procedure we describe all (homogeneous and inhomogeneous) sources of broadening with a single lineshape. Although there are more sophisticate (and more reliable) approaches to include spectral broadening, an accurate description of the lineshapes is out of the scope of this work. Nonetheless, we note that the symmetric lineshapes used here could slightly skew the visual interpretation of the spectra, because the various LH2 complexes present asymmetric lineshapes. However, such a bias is expected to be quite small, and properly accounting for the lineshape would not change the following interpretation.

As it can be seen from the tables and the graphs, the simulations give an accurate description of HL–LH2 and they reproduce the blue-shifts when moving to the other two complexes. However, the calculated shifts are underestimated; this is particular evident for HM–PucD which remains too similar to LL–LH2. As a further test, we have applied the approach used in the previous study for HL–LH2:^30^ we calculated the excitonic Hamiltonian for each of the 50 frames and we obtained the resulting exciton states and absorption spectra which were finally averaged. The resulting absorption spectra of the three complexes are shown in Fig. S3 of the ESI,† whereas the energies of the excitonic states are reported in Table S3 of the ESI.† These data show exactly the same trend as the ones obtained from the average Hamiltonian.

Before moving to a detailed analysis of the results of our calculations, it is useful to consider the experimental results on genetically modified LH2 complexes from *Rps. sphaeroides*.^21^,^55^ By constructing single (Tyr44, Tyr45 → Phe, Tyr) and double (Tyr44, Tyr45 → Phe, Leu) site-specific mutants of wild-type (WT) LH2, these studies found that the absorbance of the B850 band at 77 K was blue-shifted by about 220 cm^–1^ and 450 cm^–1^, respectively. As the single mutation corresponds to the loss of one H-bond of *β*BChl, and the double mutation to the simultaneous loss of two H-bonds (on *β* and *α*BChl), these data seem to show additivity in the H-bonds effects. However, it has to be noted that these shifts are about a half of the ones measured for LL–LH2 and PucD. It is true that the mutants refer to another organism, and that their spectra have been measured at a much lower temperature. However, from the comparison, one can conclude that the mutations localized on the H-bonding residues preserve the main excitonic characteristics of WT (HL) LH2 much more than in the low-light complexes. Moreover, it is interesting to note that the shifts in the mutants are of the same order of magnitude of those calculated for the two complexes. This consideration suggests that our calculations properly include the main effect of the H-bonds on site energies and couplings, but they miss some additional effects.

To better investigate this suggestion, in the following section we separately analyze all the possible effects (environmental, structural, *etc*) that have been proposed in the literature to explain the spectroscopic changes in the three complexes.

## 4 Dissecting the possible origins of the spectral differences

**Fig. 4:**
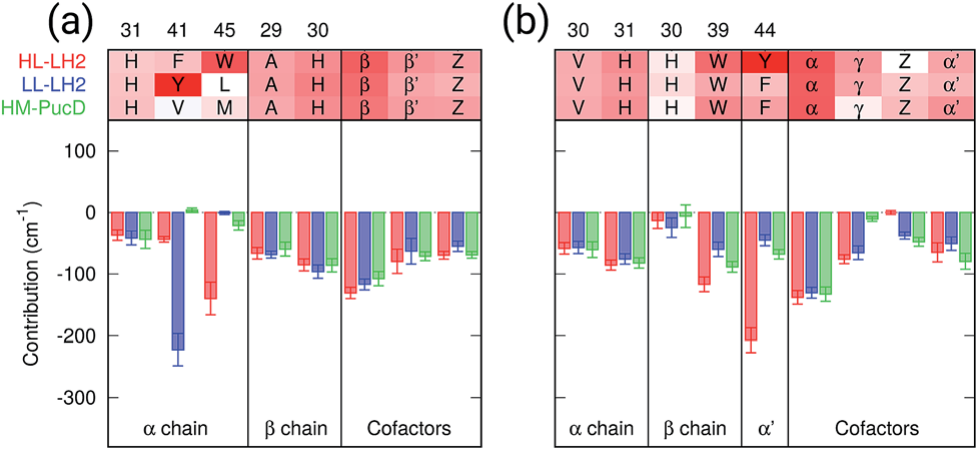
Contributions of the residues/cofactors to the *Q*_y_ excitation of *α* (a) and *β* (b) BChls in the three complexes. The average value and the standard error bar are shown; the results are computed on 20 frames of MD. Amino acids are displayed using the one letter code (H = his, F = phe, Y = tyr, V = val, W = trp, L = leu, M = met, A = ala), the two adjacent BChls in the ring are displayed using “*α*” and “*β*” whereas *Z* refers to the closest carotenoid and *γ* to the B800 BChl.

As a first analysis, we consider the effect of the composite environment (namely the protein, the membrane and the water solvent). In Fig. 4 we report the contribution of selected residues/cofactors on the *Q*_y_ excitation of the *α* and *β*BChls of the 18-meric ring in the three complexes (to have a compact notation the one letter code is used for the residues). These contributions have been obtained by repeating the calculation of the excitation energy of each BChl after “switching off” the selected residue, *i.e.*, setting to zero its charges and polarizabilities. We calculated the contribution of each residue as the difference between the excitation energy computed in the full MMPol environment and that calculated after the switch-off. A more complete analysis including all the residues within 6 Å from the BChls is shown in Fig. S4 of the ESI.†

As expected, the main contributors to the *Q*_y_ energy are the H-bonded residues, namely, Trp45 for *α*Bchl and Tyr44 for *β*BChl in HL–LH2, and Tyr41 for *α*BChl in LL–LH2. All of them lead to a red-shift ranging between 140 cm^–1^ (Trp45) to 220 (Tyr41). Notably, the effect of changing the H-bonding network from HL to LL–LH2 is not only a blue-shift for *β*BChl, due to the loss of the H-bond with Tyr44, but also a (small) red-shift for *α*Bchl, due to the replacement of Trp45 with Tyr41. The latter is in fact a stronger H-bond donor than the former.^56^ As a further interesting note, we observe that for all the BChls in the three complexes a not negligible source of red-shift is given by the adjacent BChls.

From this analysis it appears that each H-bond is responsible for a red-shift of the *Q*_y_ excitation of about 140–220 cm^–1^. In previous computational studies, different values were suggested for the same contribution, and in some cases values as large as 500 cm^–1^ were proposed.^57^ However, we have to note that those calculations were performed using a different model (not involving a polarizable environment) and different structures with respect to the ones here used.

**Fig. 5:**
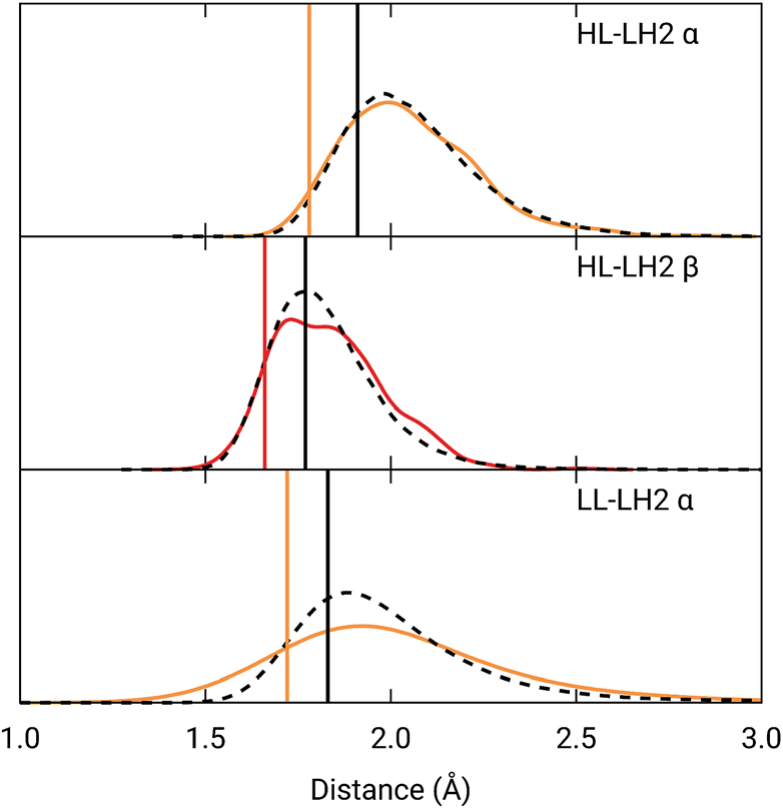
Hydrogen bond length distributions for *α*BChl–Trp45 and *β*BChl–Tyr44 of HL–LH2 and for *α*BChl–Tyr41 of LL–LH2 along the MD compared with the results from the full optimization from previous work (black sticks)^25^ and from the present constrained optimization (colored sticks) of the BChls. We reported the distribution from the whole MD (18 000 frames) as a black dashed line and from the 50 frames selected for excitonic calculations as a colored line.

To analyze the possible effect of the structures, in Fig. 5, we report the H-bond length distribution for *α*BChl–Trp45 and *β*Bchl–Tyr44 of HL–LH2 and for *α*BChl–Tyr41 of LL–LH2 along the MD trajectory, compared with the results from two differently relaxed crystal structures. The first of these relaxed structures was taken from our previous work^25^ and it was obtained at ONIOM(QM:MM) level where the BChl (without the tail) and the residues directly interacting with it, namely the axially coordinating histidine and the hydrogen-bonded residues, were included in the QM region and allowed to move. The rest of the environment was instead kept frozen at the crystal structure, including the other BChls. The second relaxed geometry (from now on indicated as “constrained optimization”) is also obtained at the ONIOM(QM:MM) level, but this time only the BChl (without the tail) was included in the QM layer. Moreover, all its dihedral angles were kept frozen at the crystal values together with the positions of all the atoms of the environment.

As it can be seen from Fig. 5, the maximum of the distribution of the H-bond distances for the three investigated pairs agrees well with a fully relaxed structure while the constrained relaxation (which remains closer to the original crystal structure) gives somehow shorter distances. Nonetheless, the differences are around 0.1 Å and their effects on the induced shift on the *Q*_y_ excitation are less than 40 cm^–1^.

**Fig. 6:**
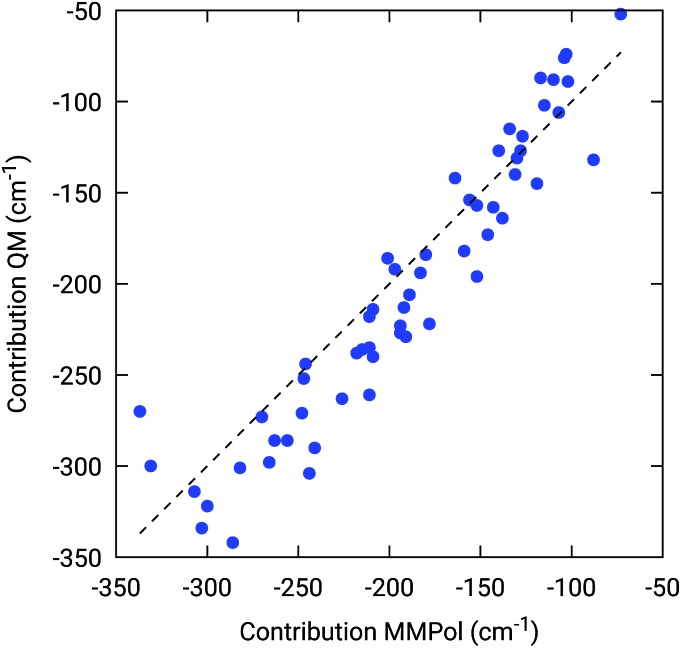
Benchmark of the MMPol description of the H-bonding residue against a QM description. The measured property is the effect of the residue (Tyr or Trp) on the BChl *Q*_y_ excitation energy. For this test, 20 structures extracted from the MD trajectory were used.

To further confirm the robustness of our results on the description of H-bond effects, we have performed two tests. In the first test, we have validated the accuracy of the selected MMPol parameters (charges and polarizability). To do so, we have compared the H-bond contributions reported in Fig. 4 with the ones computed on the same 20 structures extracted from the MD trajectory, this time using a QM description also for the H-bonded residue under investigation, while leaving the rest of the environment at MMPol level. The correlation between the QM/MMPol and the QM/QM/MMPol results are reported in Fig. 6.

The good correlation tells us that in the present system, the H-bond effects are well described by a classical model including both electrostatic and polarization effects, whereas possible non classical effects (dispersion and/or charge-transfer) are negligible.

In the second test, we assess the quality of the selected DFT functional in describing the H-bond effects on the *Q*_y_ excitation. For this test we have repeated the calculations of the QM/MMPol H-bond contributions with five different functionals selected among the most successful ones to describe electronic excitations in large chromophores (PBE0, CAM-B3LYP, M062X, LC-BLYP, *ω*B97XD). The correlations between B3LYP and these functionals are reported in Fig. S5 of the ESI.† The obtained results show an almost perfect correlation with PBE0, while all the other (long-range corrected) functionals give a larger spread of values, the maximum differences being for *ω*B97XD. Thus, the sensitivity of the excitation energy to the H-bond increases with the amount of exact exchange in the DFT functional. However, there is no systematic bias between one functional and another, and on average the results are quite consistent.

All these tests confirm that our description of the H-bond effects on the *Q*_y_ excitation energy is sufficiently robust.

The changes in the H-bonding network observed in the three LH complexes also correspond to changes in the acetyl dihedral angle. As already commented in the Introduction, the acetyl (C=O) double bond is conjugated with the macrocycle ring: we thus expect that moving the acetyl out of the macrocycle plane could lead to a blue-shift of the *Q*_y_ excitation, and indeed this effect has been proposed as responsible for the spectroscopic changes observed from HL to LL–LH2.^18^

**Fig. 7:**
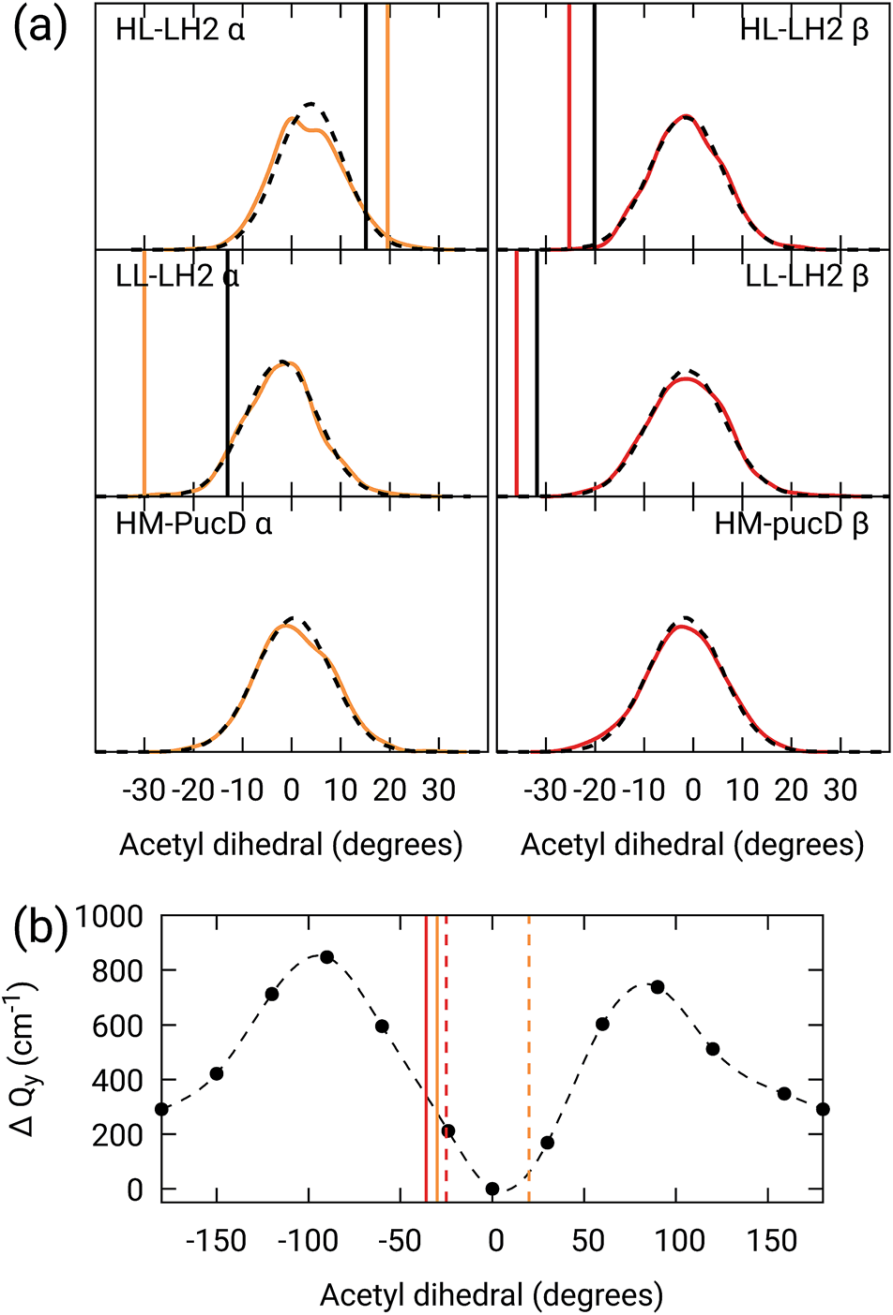
(a) Dihedral angle distributions from the MD trajectory compared with the results from the previous work full optimizations (black sticks)^25^ and from the present work constrained optimizations (colored sticks). We reported the distribution from the whole MD (18 000 frames) as a black dashed line and the one from the 50 frames selected for QM calculations as a colored line. (b) Blue-shift of the excitation energy along a relaxed scan of the LL–LH2 *β*BChl acetyl dihedral. The vertical sticks show the dihedral angles of the crystal structures: *α*BChls yellow, *β*BChls red, HL–LH2 dashed line, LL–LH2 solid line.

As done for the H-bond distances, also here we compare the distribution for the dihedral angles of *α* and *β*BChl in the three different complexes along the MD trajectory with the results from the two differently relaxed crystal structures. This comparison, reported in the top panel of Fig. 7, clearly shows that for all the investigated complexes, the calculated distributions are centered on a planar structure for both *α* and *β*BChl. This finding disagrees with what found in the constrained optimizations, which, we recall, coincide with the crystal data for all dihedral angles. The latter in fact indicate a dihedral angle of *ca* ±20° for *α* and *β*BChl of HL–LH2, which changes to –30° in LL–LH2 (no crystal data are available for PucD). The out of plane rotation of the acetyl group is also confirmed by the full optimizations of the BChls obtained in the previous work, even though for *α*BChl in LL–LH2 the rotation is smaller.

To check the origin of the planarization in the MD simulations, we run an optimization of the BChls described at the same MM level within a frozen environment. Also in this case, the acetyl group planarizes (all the dihedral values are reported in Table S4 of the ESI†), so we can conclude that the MM force-field here used for the BChl overstabilizes planarity in the acetyl orientation. This has the consequence of reducing the differences in the site energies of the different complexes, as their BChls present roughly the same planar acetyl orientation.

**Fig. 8:**
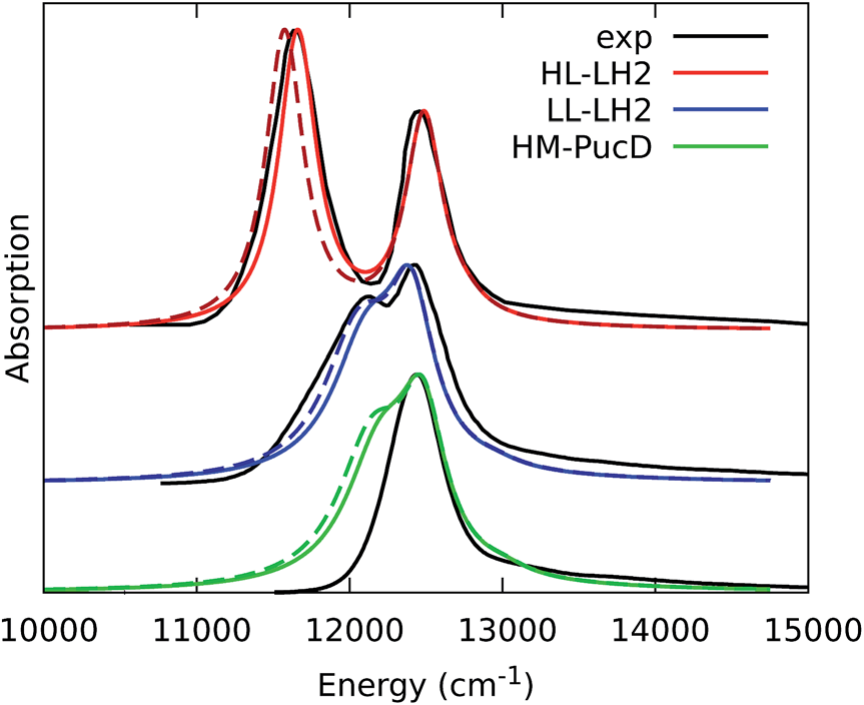
Comparison between simulated and experimental absorption spectra of the three investigated complexes. The spectrum of HL–LH2 is the same reported in Fig. 3 while the spectra of LL–LH2 and HM–PucD have been recalculated by correcting the site energies of *α*BChl and *β*BChl by the additional blue-shift induced by the different out-of-plane distorsion as predicted by crystal data (for HM–PucD we have assumed the same distorsion as LL–LH2). The spectra computed with the inclusion of CT states are drawn as dashed lines.

To quantify the real effect of the torsion of the acetyl group on the *Q*_y_ excitation, we performed a relaxed scan of the *β*BChl geometry within the LL–LH2, and finally calculated the *Q*_y_ excitation energy on the corresponding structures. This scan has been obtained at ONIOM(B3LYP/MM) level where the QM subsystem is made of the *β*BChl together with the coordinating histidine and the close by phenylalanine while the MM subsystem (the phytyl chain of the BChl, the other BChls and cofactors and the protein) is kept frozen in the crystal configuration. For each torsional angle, the QM subsystem has been allowed to relax. From the results reported in the right panel of Fig. 7, we can obtain a rough estimate of what we are missing in our MD descriptions, where the acetyl dihedral angle always averages to zero. If we assume valid the crystallographic estimates of the dihedral angles, a further blue-shift of about 220 cm^–1^ should be considered for the *α*BChl (for which the crystal dihedral angle changes from 20° to –30°) and 120 cm^–1^ for the *β*BChl (for which the dihedral angle change from –25° to –36°). We note that if we add these shifts to the *Q*_y_ energies calculated from the MD and combine them with the corresponding couplings (the dihedral angle in fact does not significantly affect the coupling) we obtain that the energy differences for the *k* = ± 1 exciton between LL and HL–LH2 increase to 452 cm^–1^ (486 cm^–1^ if we also include the CT effect). These results, compared with the experimental shift of 501 cm^–1^, seem to show that an accurate prediction of the dihedral angle could be the missing piece in our simulation to fully reproduce the spectroscopic differences between HL and LL forms of LH2. This analysis is exemplified in Fig. 8, where we report the same comparison reported in Fig. 3, but now the simulated spectra of LL–LH2 and HM–PucD have been obtained by correcting the site energies by the additional blue-shift induced by the different out-of-plane distorsion of *α*BChl and *β*BChl as predicted by crystal data (for HM–PucD we have assumed the same distorsion as LL–LH2).

This conclusion in some way goes against what reported in recent computational studies by De Vico and coworkers,^20^,^58^ who used a multistate multiconfiguration restricted active space with second-order perturbation theory correction (MS-RASPT2) to calculate the excitation energies of the BChl at their crystal structure. In the same study, instead, a different source of the blue shift was suggested, that is, the change in the BChl macrocycle ring curvature. In particular, by using the geometry of *α* and *β*BChls taken directly from the crystal structure (without any relaxation) of HL and LL–LH2 huge blue-shifts of the order of 0.23 eV (*ca.* 1850 cm^–1^) are found for both BChls.

In order to test this hypothesis, we have compared the unrelaxed crystal structures with the two sets of refinements of the same crystal structures already considered in the previous analysis, namely, (i) the ones obtained from a constrained optimization of the BChls where all their dihedral angles were kept frozen to the crystal values, and (ii) the full optimization where all the internal degrees of freedom of the BChl and of the close-by residues were allowed to relax.

The obtained results, reported in Table S5 of the ESI,† clearly show that, if we use the crystal structures without any relaxation, a very large (and unphysical) blue-shift of *ca.* 2000 cm^–1^ is found for both *α* and *β*BChls when moving from LHL to LL, exactly as found by De Vico *et al.*^20^ However, as soon as we relax the bond lengths (and bond angles), still keeping the dihedral angles frozen (and hence the macrocycle curvature), these differences almost disappear: they reduce to 40 cm^–1^ and 60 cm^–1^ when calculated for the isolated *α* and *β*BChls, respectively, and to 65 and 186 cm^–1^ when the effect of the MMPol environment is included. If we further relax all the internal degrees of freedom together with the close by residues, we do not see any further significant change, showing that the bond lengths play a major role in determining the excitation energy. As a matter of fact, this result was expected due to the conjugated nature of the macrocycle ring.

To reach a more detailed picture of the role of bond lengths in the excitation energy shifts, we trained a linear regression model, using all bond lengths in the macrocycle ring as explanatory variables for the *Q*_y_ excitation energies computed along the MD trajectory. As shown in Fig. S7,† the prediction based on bond lengths explains more than 60% of the variability in excitation energies. Therefore, bond lengths are the main factor determining *Q*_y_ excitation energies of BChls in LH2. We then employed the parameters of the linear regression to predict the excitation energies in crystal and optimized structures. These structures were not employed for the fitting, but nonetheless their excitation energy is consistently predicted by the bond lengths model. In particular, this model correctly predicts that both α and β unrelaxed structures of HL–LH2 are strongly red-shifted. We can thus conclude that the bond lengths are ultimately responsible for the unphysical excitation energy in the HL–LH2 ; 1NKZ crystal structure, which also explains the huge blue-shift found for both *α* and *β*BChls when moving from HL to LL.

These data, together with the ones obtained from the MD (where the macrocycle ring is almost planar), indicate that the curvature of the macrocycle as described by the crystal structures does not significantly contribute to the blue-shift of the *Q*_y_ excitation energies. In addition, this analysis clearly shows the limitations of the crystal data for bond lengths (and angles) especially when a conjugated pigment is involved.

As a last analysis, we considered the effect of higher energy CT states between adjacent BChls, which can couple with the *Q*_y_ excitations and finally lead to changes in the exciton energies.

To do that, we have calculated the four *Q*_y_–CT couplings in the inter-chain and intra-chain BChl dimers of HL and LL–LH2 along the respective MD trajectories. Due to the very close similarity observed up to now in the simulation of LL–LH2 and PucD, this analysis of CT has been limited to the former complex only.

**Fig. 9:**
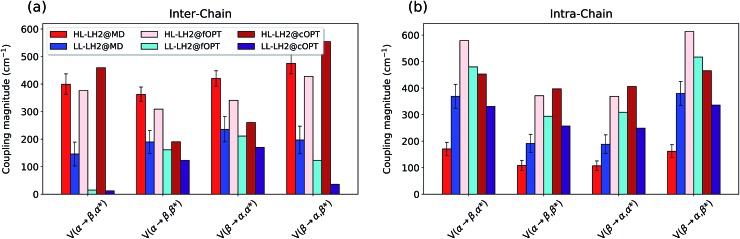
Comparison of the *Q*_y_–CT couplings in the (a) inter-chain and (b) intra-chain BChl dimers along the MD trajectory and for the crystal structures. The results of the previous work^25^ are marked as fOPT, whereas cOPT refers to the results of the constrained optimizations. Absolute values are shown (cm^–1^). Error bars indicate 95% confidence intervals.

As shown in Fig. 9, the present results seem to indicate that the effect of CT couplings is more modest than estimated in our previous study where the effects of the fluctuations were not included.^25^ Within that static picture, the *Q*_y_–CT couplings were systematically smaller for LL–LH2 with respect to HL–LH2, with some of the inter-chain couplings dropping to almost zero. However, when averaging these couplings along the MD trajectory, the differences between the two systems are reduced, even though the LL–LH2 inter-chain couplings are significantly smaller than those for HL–LH2. For the intra-chain couplings, instead, the picture obtained with MD is reversed with respect to the static picture (Fig. 9b), and the couplings in LL–LH2 become larger than those of HL–LH2.

In order to ascertain that these differences do not arise from some geometrical bias of the BChls, we recomputed the *Q*_y_–CT couplings using the geometries from the constrained optimization described before: these calculations (see cOPT values in Fig. 9) confirm the same picture obtained in the previous work (fOPT values) showing that the differences found in the couplings from MD structures are due to the different average configuration of the 18-meric ring (and of the embedding protein) with respect to the crystal structure. In fact, the results obtained along the MD trajectory stem from the variety of distances and orientations undertaken by the BChl dimers, which, combined with the sensitivity of CT couplings to the geometry,^44^ give rise to a large variability of coupling values (see also the average values and the standard deviations reported in the Table S2 of the ESI†). Notably, the variance of *Q*_y_–CT couplings is larger in LL–LH2 than in HL–LH2, possibly as a consequence of the larger geometrical freedom of the *β*BChl. As a final note we observe that the presence of dark states (identified as charge transfer states and/or polaron pairs) have been also investigated experimentally in the HL–LH2 and PucD forms from *Rps. palustris* using two-dimensional electronic spectroscopy.^59^ These experimental observations seem to suggest that such states are present in both complexes and they can act as strong quenchers. However, in those observed dark states likely refer to relaxed states, whose energy is much more red-shifted than what found in the present simulations where we have calculated the vertical CT states.

## 5 Why is PucD so blue-shifted?

From the previous analysis, it came out that by removing the H-bond connecting the BChls of two different units, a significant decrease in the coupling is obtained. We have explained these effects in terms of a much larger distribution of the relative orientations explored by the inter-chain BChls in LL–LH2 and PucD complexes with respect to HL–LH2, which finally averages in smaller couplings between different units. On the contrary, the coupling between BChls of the same unit has shown to stay almost the same even when one (LL–LH2) or both (PucD) H-bonds are removed. One could explain this findings saying that the dynamics of the αβ chains of the same unit preserve the relative orientation of the BChls which are anchored to the respective chains through His residues, and the change in H-bonds has only a minor effect.

**Fig. 10:**
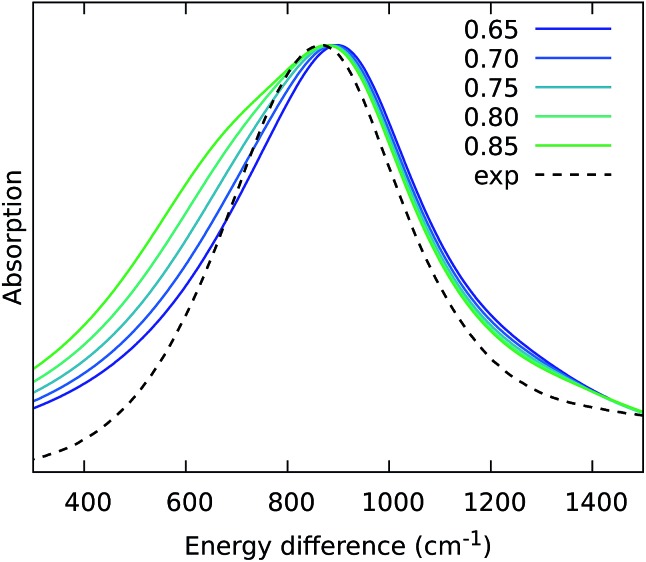
Simulated spectra of PucD models obtained for different scaling factors of the intra-chain coupling *V*2αβ. The *x*-axis corresponds to the energy shift with respect to the B850 peak of HL–LH2.

However, the validity of this assumption cannot be fully validated by our MD simulations. In fact, if a complete release of all H-bonds interactions between the BChls and the binding chain (as it happens in PucD) would lead to changes in the relative position of the BChls, this could be seen only allowing the complex to explore structures which are farther with respect to the starting (crystal) structures. In particular, the intra-chain pair could be more flexible than what revealed in our MD simulation, thus leading to a further reduction of the corresponding couplings. If this is the case, we would see a significant effect in the PucD. To have an indirect check of this suggestion, we have estimated how much the intra-chain couplings should be reduced to achieve the experimental shift: to do that we have recalculated the position of the *k* = ±1 exciton state of PucD using the excitonic parameters obtained from our simulation and scaling only the intra-chain coupling *V*2αβ by factors ranging from 0.65 to 0.85. The results are reported in Fig. 10. As it can be seen from the graph, by reducing the *V*2αβ of 30% we get the expected spectrum showing a single band at about 800 nm. We note however, that here we have also corrected the Qy excitations of *α* and *β*BChls so to account for the artificial planarization of the acetyl group already seen in LL–LH2. If this further effect is not included, we need to introduce a much larger scaling of the coupling (namely around 50%) which would be rather unlikely.

## 6 Conclusions

In this work, we have investigated the origin of the exciton tuning in LH2 complexes when grown in different light conditions. The study has been made possible by the combination of molecular dynamics simulations and excitonic Hamiltonians calculated through a multiscale QM/MMPol approach. In particular, we studied the high light and low light forms of LH2 from *Rps. acidophila* and a third structure encoded by the *puc*BA_d_ gene from *Rps. palustris*. Whereas for the first two structures a high-resolution crystal structure is available, for the third one it is not, so we employed the homology modeling technique to get a starting structure for the molecular dynamics. By applying the same computational strategy to the three complexes, we could achieve a detailed understanding of the origin of their excitonic and spectroscopic differences.

First, we investigated the role of the composite environment (the protein, the membrane and the solvent) in the tuning of the *Q*_y_ excitations of the BChls and of their electronic couplings. For the excitation energies, we found solid evidence that the electrostatic and polarization effects of the environment remain similar in the three complexes. Instead, the differences in the H-bonding residues explain a large part of the observed exciton tuning. In particular, we have estimated that the loss of each H-bond not only accounts for about 200 cm^–1^ of blue-shift of *Q*_y_ but it also indirectly affects the electronic couplings. Specifically we have seen that because the *β*BChl in HL–LH2 is connected to the neighboring unit by a H-bond, the thermally induced fluctuations in its orientation remain correlated to the one of the adjacent *α*BChl in such unit. In the other two complexes, instead, the H-bond is lost and the orientation in the two BChls belonging to different units becomes uncorrelated thus significantly reducing their coupling.

We also investigated the role of the internal geometry of the pigments, through the acetyl torsion and the curvature of the macrocycle ring of the BChls. For the first parameter, we found that, as expected, a deviation from the planarity results in a blue-shift. Indeed, this effect, when combined with all the above described H-bond effects on site energies and couplings, could fully explain the measured spectral differences between HL and LL forms of LH2. On the contrary, the curvature of the macrocycle ring seems not to play a major role when a proper description of the bond lengths within the conjugated ring is accounted for. In particular, the huge differences found in the literature for the *Q*_y_ excitations of HL and LL–LH2 (ref. 20,58) can be accurately explained by the fact that the crystal (; 1NKZ) structure of HL–LH2 displays an unphysical conjugation pattern of the macrocycle ring due to inaccurate bond lengths. Instead, the BChls from the crystal structure of LH3 (; 1IJD) present a more regular pattern of bond lengths within the macrocycle ring and, as a result, a huge blue-shift is obtained when compared to the HL–LH2.

Furthermore, we investigated the role of higher energy CT states between adjacent BChls in the 18-meric ring which can couple to the *Q*_y_ excitations and finally affect the excitons. Indeed, we have found that the HL and LL forms of LH2 have different effects of CT states, but also that thermal fluctuations tend to reduce these differences with respect to a picture based on the crystal structure.^25^

Finally, we have suggested a possible explanation of the measured large change in the spectrum when moving from LL–LH2 to PucD in terms of an additional reduction of the couplings, this time involving the BChls belonging to the same unit. What is difficult to say from the present simulations is whether this further decrease of the couplings in PucD involves distortions of the 18-meric ring with respect to LL–LH2 or is instead induced by a larger mobility of the BChls around their unaffected average position. In order to confirm one or the other of the two effects would in fact require to largely extend the time windows to be investigated by the MD trajectory. This is certainly an aspect which requires further investigation both from an experimental and a computational point of view.

## Conflicts of interest

There are no conflicts to declare.

## Supplementary Material

Supplementary information
