# Supplementary material for: The molecular mechanisms of light adaption in light-harvesting complexes of purple bacteria revealed by a multiscale modeling
Source: Chem Sci. 2019 Sep 27;10(42):9650–62. doi: 10.1039/c9sc02886b (PMC6988754; doi:10.1039/c9sc02886b)
Supplement: Supplementary file 1 [file SC-010-C9SC02886B-s001.pdf]

Electronic Supplementary Material (ESI) for Chemical Science.
This journal is © The Royal Society of Chemistry 2019

Electronic Supplementary Information (ESI) The molecular mechanisms of light adaption in light-harvesting complexes of purple bacteria revealed by a multiscale modeling

Felipe Cardoso Ramos, Michele Nottoli, Lorenzo Cupellini, and Benedetta Mennucci*

Dipartimento di Chimica e Chimica Industriale, University of Pisa, via G. Moruzzi 13, 56124, Pisa, Italy

E-mail: benedetta.mennucci@unipi.it

S1 Additional figures

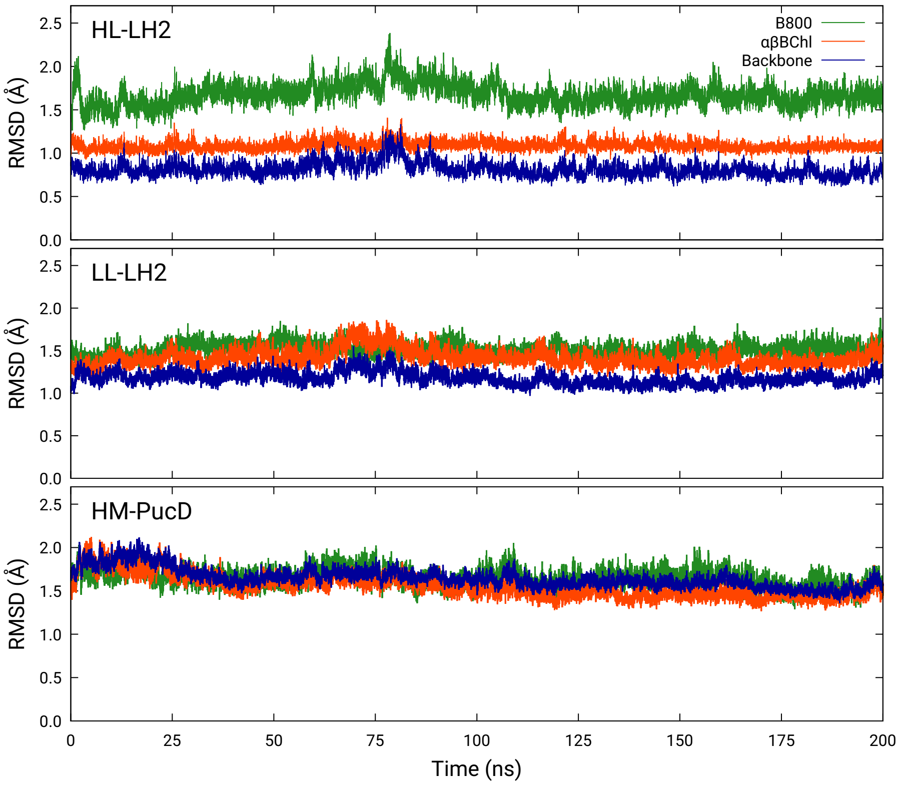

Figure S1: RMSD plot for the backbone of LH2 α-helices (blue), α and βBChl (orange) and B800 (green), with respect to the crystal structures (or Homology Modelling structure for HM-PucD). The selected atoms from BChls were the same as the QM part in the multiscale analysis (See Fig. 1 from the main text).

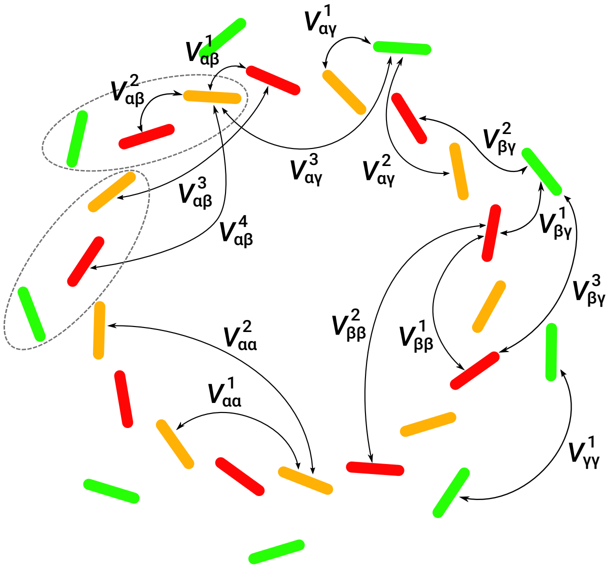

Figure S2: Schematic representation of the electronic coupling definitions. The BChls are highlighted using the following color code: yellow for αBChl, red for β and green for B800 (for brevity defined as γ). The superscript ranks the proximity according to inter-pigment distance. The dashed ellipses highlight two subunits of the protein.

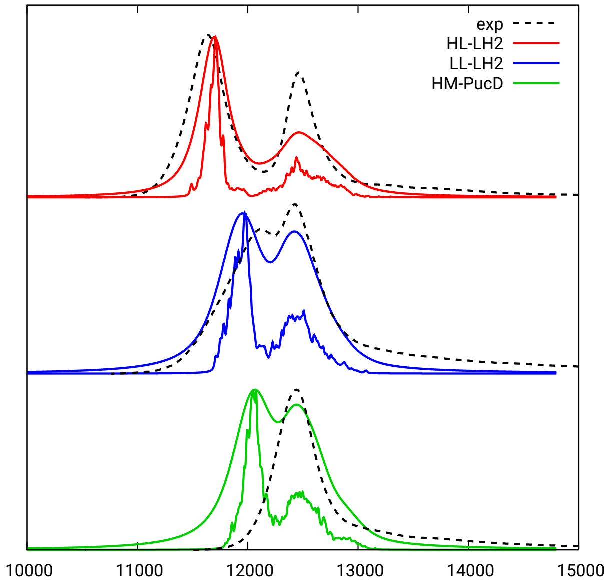

Figure S3: Plot showing the sum of stick spectra computed on individual frames of molecular dynamics, their convolution obtained with Lorentzian functions and the experimental spectra (as black dashed lines). The computed spectra are shifted by -1247 cm^{-1} to match the experimental B800 band. Moreover, we used the procedure described in Ref. 1 to remove the inhomogeneous broadening from the internal degrees of freedom of the BChls.

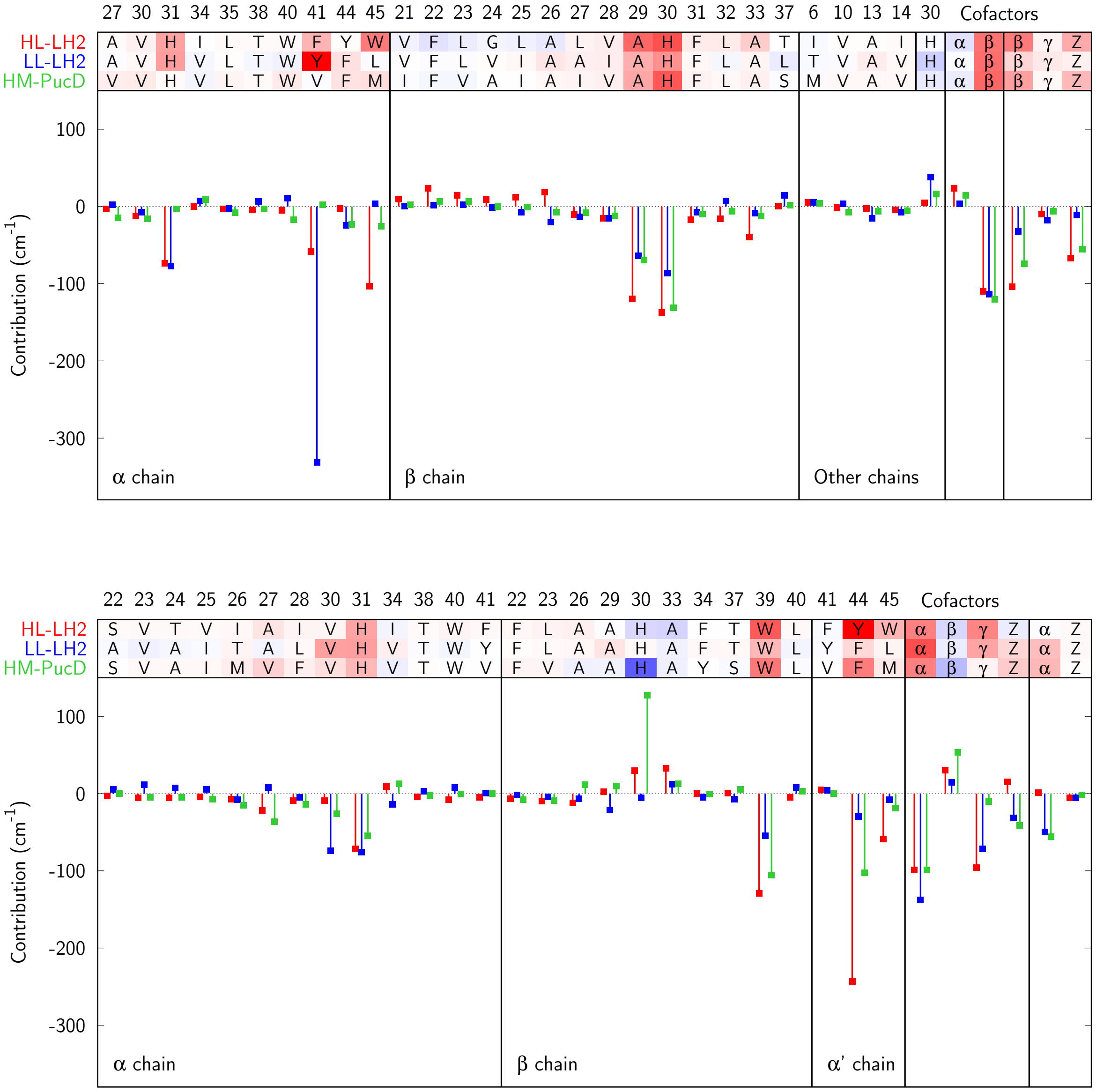

Figure S4: Contributions to the BChl transitions from all the residues within 6 Å from them (top: αBChl, bottom: βBChl).

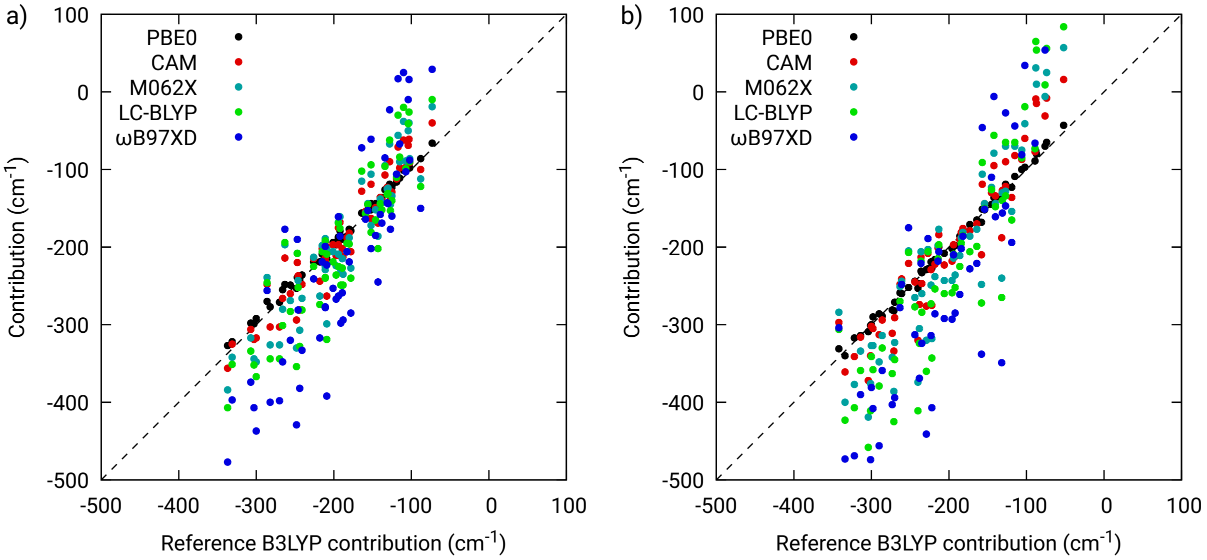

Figure S5: Benchmark of several different functionals against the B3LYP results. The measured property is the effect of the H-bonding residue on the BChl Q_y excitation energy. The results are computed on 20 structures extracted from the MD trajectory. The plot a) refers to calculations in which the H-bonded residue is treated at the MMPol level whereas the plot b) refers to calculations in which the residue is treated at QM level.

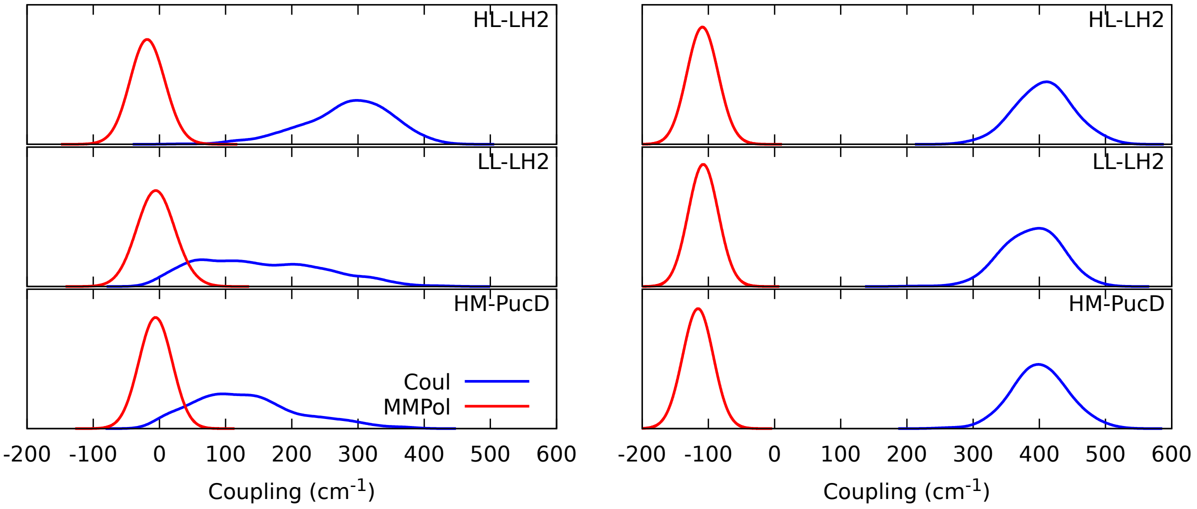

Figure S6: Distributions of the coupling components along the MD trajectory, left: $V^1_{\alpha\beta}$, right: $V^2_{\alpha\beta}$. The distribution of the Coulomb component (V_{Coul}) is drawn in blue, that of the environmental component (V_{MMPol}) in red.

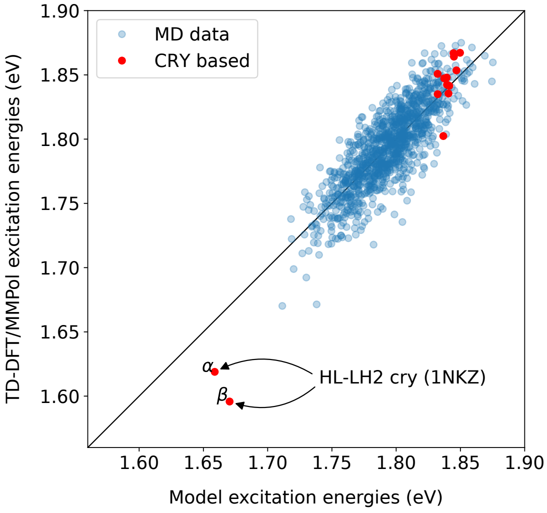

Figure S7: Results of the regression of Q_y excitation energies using the bond lengths of the macrocycle ring ($R^2_{\text{adj.}} = 0.68$). Blue dots represent excitation energies calculated/predicted on the MD structures, and used to fit the model. Red dots represent all the crystal-based structures (including the full and the constrained optimization structures), which were not included in the fit, and are here used as a test sample. The outliers indicated by the arrows correspond to the unrelaxed crystal structures of HL-LH2. All excitaton energies have been computed from the isolated BChls with geometries coming from the MD and the crystal structures

S2 Additional tables

Table S1: Average of the equivalent couplings and standard deviations computed along 50 frames of MD. A schematic representation of the nomenclature is shown in Fig. S2. All the missing couplings are between distant BChls and are treated as 0 cm^{-1}.

Type	HL-LH2		LL-LH2		HM-PucD	
	V (cm^{-1})	σ (cm^{-1})	V (cm^{-1})	σ (cm^{-1})	V (cm^{-1})	σ (cm^{-1})
$V^1_{\alpha\beta}$	266	55	149	89	127	74
$V^2_{\alpha\beta}$	298	35	281	37	285	35
$V^3_{\alpha\beta}$	18	2	14	4	12	3
$V^4_{\alpha\beta}$	17	1	15	2	15	2
$V^1_{\alpha\alpha}$	-62	5	-66	7	-66	6
$V^2_{\alpha\alpha}$	-9	1	-9	1	-9	1
$V^1_{\beta\beta}$	-48	6	-30	12	-22	10
$V^2_{\beta\beta}$	–	–	-5	2	-3	1
$V^1_{\alpha\gamma}$	38	21	36	4	35	4
$V^2_{\alpha\gamma}$	-15	13	-13	2	-13	2
$V^3_{\alpha\gamma}$	-2	2	-6	2	-5	2
$V^1_{\beta\gamma}$	-8	4	-4	6	-4	5
$V^2_{\beta\gamma}$	-5	8	-1	3	1	3
$V^3_{\beta\gamma}$	9	7	8	1	8	1
$V^1_{\gamma\gamma}$	-34	25	-29	4	-28	4

Table S2: Top: Average of the equivalent charge transfer state energies and standard deviations. Bottom: Average of the equivalent charge transfer couplings and standard deviations. These data are computed along 10 frames of MD

Type	HL-LH2		LL-LH2	
	E (cm^{-1})	σ (cm^{-1})	E (cm^{-1})	σ (cm^{-1})
E^1_{CT} ($\alpha \to \beta$)	20846	1460	23538	2189
E^1_{CT} ($\beta \to \alpha$)	21790	1539	20724	1958
E^2_{CT} ($\alpha \to \beta$)	22412	1606	22783	2063
E^2_{CT} ($\beta \to \alpha$)	23752	1401	22059	1924
Type	V (cm^{-1})	σ (cm^{-1})	V (cm^{-1})	σ (cm^{-1})
$V^1_{CT}(\alpha \to \beta, \alpha^*)$	399	165	146	198
$V^1_{CT}(\alpha \to \beta, \beta^*)$	363	233	190	206
$V^1_{CT}(\beta \to \alpha, \alpha^*)$	420	236	236	240
$V^1_{CT}(\beta \to \alpha, \beta^*)$	475	171	197	219
$V^2_{CT}(\alpha \to \beta, \alpha^*)$	170	119	369	165
$V^2_{CT}(\alpha \to \beta, \beta^*)$	108	156	191	215
$V^2_{CT}(\beta \to \alpha, \alpha^*)$	107	150	188	216
$V^2_{CT}(\beta \to \alpha, \beta^*)$	162	113	380	169

Table S3: Bright excitonic states (cm^{-1}) computed from the average excitonic Hamiltonian and from average over spectra (Fig. S3). The values reported in parenthesis are computed with the inclusion of CT states.

	HL-LH2	LL-LH2	HM-pucD
Average Hamiltonian			
k= ±1	12906 (12821)	13183 (13133)	13281 (13230)
k= ±8	14004 (14000)	14039 (14032)	14091 (14086)
B800	13734 (13733)	13649 (13649)	13724 (13724)
Average over spectra			
k= ±1	12908	13151	13262
B800	13660	13619	13641

Table S4: Values of the acetyl dihedral angle for different structures (crystal structure Cry, full optimization fOpt and MM optimization MM-Opt).

	BChl	Cry	fOpt	MM-Opt
HL-LH2	α	20	15	8
	β	-25	-20	-5
LL-LH2	α	-30	-13	-6
	β	-36	-32	-6

Table S5: Site energies (cm^{-1}) from constrained geometry optimization. The values in parentheses refer to calculations without the effect of the MMPol.

	α	β
Structure	HL	
Cry	12253 (13057)	12010 (12871)
cOpt	14050 (14833)	13865 (14658)
fOpt	13978 (14800)	13954 (14853)
Structure	LL	
Cry	14325 (15036)	14127 (14804)
cOpt	14123 (14874)	14034 (14426)
fOpt	14032 (14859)	14154 (14899)

References

(1) Cupellini, L.; Jurinovich, S.; Campetella, M.; Caprasecca, S.; Guido, C. A.; Kelly, S. M.; Gardiner, A. T.; Cogdell, R.; Mennucci, B. *J. Phys. Chem. B* **2016**, *120*, 11348–11359.
